# Patient Perceptions About Opioid Risk Communications Within the Context of a Randomized Clinical Trial

**DOI:** 10.1001/jamanetworkopen.2022.27650

**Published:** 2022-08-18

**Authors:** Abby R. Dolan, Erica B. Goldberg, Carolyn C. Cannuscio, Matthew P. Abrams, Rachel Feuerstein-Simon, Xochitl Luna Marti, Jason Mazique, Marilyn M. Schapira, Zachary F. Meisel

**Affiliations:** 1 Center for Emergency Care Policy and Research, University of Pennsylvania, Philadelphia; 2 Center for Public Health Initiatives, University of Pennsylvania, Philadelphia; 3 Williams College, Williamstown, Massachusetts; 4 Center for Health Equity and Research Promotion, Philadelphia VA Medical Center, Philadelphia, Pennsylvania; 5 Penn Injury Science Center, University of Pennsylvania, Philadelphia

## Abstract

**Importance:**

Opioid overdose rates continue to increase, and extant literature suggests that many individuals who use heroin were first introduced to opioids through a medical prescription.

**Objective:**

To explore patient experiences related to decisions regarding analgesia after an emergency department visit within the context of a randomized clinical trial aimed to test the efficacy of risk communication interventions on treatment preference, risk recall, and use of opioids.

**Design, Setting, and Participants:**

This qualitative study of 36 patients making decisions regarding analgesia included qualitative interviews with participants in 2 risk intervention groups. Interviews were audio recorded, transcribed, and edited to remove identifying information to protect the confidentiality of participants. Interviews were conducted from June 4, 2019, to August 6, 2019. We conducted thematic analysis from August to December 2019 using a mixed inductive and deductive approach. Participants received $20 in compensation. The study was conducted in 4 geographically diverse emergency departments in the United States. Participants were adults presenting to the emergency department with either musculoskeletal back or neck pain or kidney stone–related pain. Eligibility criteria included being aged 18 to 70 years, capable of providing informed consent, English speaking or having English comprehension, eligible for emergency department discharge within 24 hours of enrollment, and able to access email or a smartphone.

**Interventions:**

Participants enrolled from the main randomized clinical trial received 1 of 2 risk interventions: a probabilistic opioid risk tool or a narrative-enhanced probabilistic risk tool (ie, participants viewed eight 1- to 3-minute short videos of patients discussing their experiences with pain treatment and positive and negative experiences with opioid use).

**Main Outcomes and Measures:**

Factors reported by participants to have influenced their decision-making regarding acute pain and treatment.

**Results:**

Thirty-six participants were interviewed, 18 in the group who received the probabilistic risk tool alone and 18 in the group who received the additional narrative-enhanced probabilistic risk tool intervention. The median age was 38 years (range, 21-67 years), 22 individuals were female (61%), 14 were Black or African American (39%), and 14 were White (39%). Five themes emerged from the analysis in the following domains: the factors associated with the risk interventions; clinician paternalism; analgesia attributes and previous experiences; individual self-identity, attitudes, and values; and perceptions of clinician bias.

**Conclusions and Relevance:**

Most participants commented on the powerful lessons they learned from the risk interventions. More research is needed to understand how patients incorporate risk information into their decision-making process.

## Key Points

**Question:**

What can be learned about patient experiences related to decisions regarding analgesia after an emergency department encounter within the context of a randomized clinical trial testing the efficacy of risk communication interventions?

**Findings:**

For the 36 patients interviewed for this qualitative study, patient decision-making regarding pain treatment in the emergency department was associated with risk interventions, clinician opinion, patients’ previous experiences with pain treatment, their values and perceptions of self, and experiences of bias.

**Meaning:**

The clinical role of narrative-enhanced, risk-informed communication for acute pain management in acute care settings is important and should be considered along with other factors patients consider in the decision-making process.

## Teaser

This qualitative study investigates patient decision-making regarding emergency department pain treatment after risk interventions associated with opioid use.

## Introduction

The overdose crisis claimed more than 100 000 lives in the United States in the 12 months leading up to April 2021.^1^ The majority of these overdoses were opioid related.^2^ Approximately 80% of individuals who use heroin were first introduced to opioids through a medical prescription.^3^ In response, policy makers and health care systems have attempted to reduce opioid prescribing as a way to prevent individuals from transitioning to using illicit substances.^4,5,6,7^ However, in recent years, chronic pain advocates and researchers have discovered the response has been unbalanced and has led clinicians to sometimes underprescribe appropriate analgesic medications, leaving patients with undertreated pain as well as resulting in significant racial disparities in prescribing.^8,9,10,11^

The Life Stories for Opioid Risk Reduction in the ED (Life STORRIED) clinical trial was a Patient-Centered Outcomes Research Institute (PCORI)–funded randomized clinical trial (RCT) conducted to understand how to communicate risks related to opioid prescriptions while placing some of the decision-making power back into the hands of patients.^12^ The aim of this trial was to engage patients who presented to the emergency department (ED) with either musculoskeletal back or neck pain or renal colic in a shared decision-making process incorporating their values and preferences into their pain management decisions after their ED visit. Presenting quantitative risk information in an engaging and easy-to-understand format is challenging, and the value of using narratives to communicate this information is still largely unknown.^13^ Therefore, we sought to add to this knowledge base.

In the Life STORRIED trial, participants were randomly assigned to 1 of 3 groups, 2 of which were risk interventions. Detailed methods of the RCT follow. The goal of this qualitative study was to understand how the risk interventions affected patients’ decision-making processes for pain treatment in the context of this trial; broadly, the study team was interested in how participants reflected on their experiences and how this shaped their perceptions regarding treatment. A prior study conducted with patients in the ED indicated that adverse effects of treatment options and a patient’s experiences with medications are often topics of discussion between clinicians and patients making decisions about pain treatment.^14^ We were interested in understanding what additional factors were associated with this decision-making process.

## Methods

This study was approved by the University of Pennsylvania institutional review board, which served as the institutional review board of record for the Northwell Health site, and the University of Alabama and Mayo Clinic institutional review boards. Participants provided informed consent verbally over the telephone and received $20 in compensation.

### RCT Methods

In the Life STORRIED trial, participants were recruited from 4 acute care settings: Philadelphia, Pennsylvania (urban); Long Island, New York (suburban); Rochester, Minnesota (rural); and Birmingham, Alabama (urban). Race and ethnicity were self-reported by participants. Eligibility criteria included being aged 18 to 70 years; capable of providing informed consent; having a chief complaint indicating acute neck or back pain, flank pain, or both; English speaking or having English comprehension; eligible for ED discharge within 24 hours of enrollment; and able to access email or a smartphone. Participants were randomly assigned to 1 of 3 groups. All participants received a general risk information sheet with instructions on how to care for the relevant condition at home and information about the risks of different types of pain treatment. For 1 of the 3 groups, this was the only information received because this group represented usual care.

**Figure 1.**
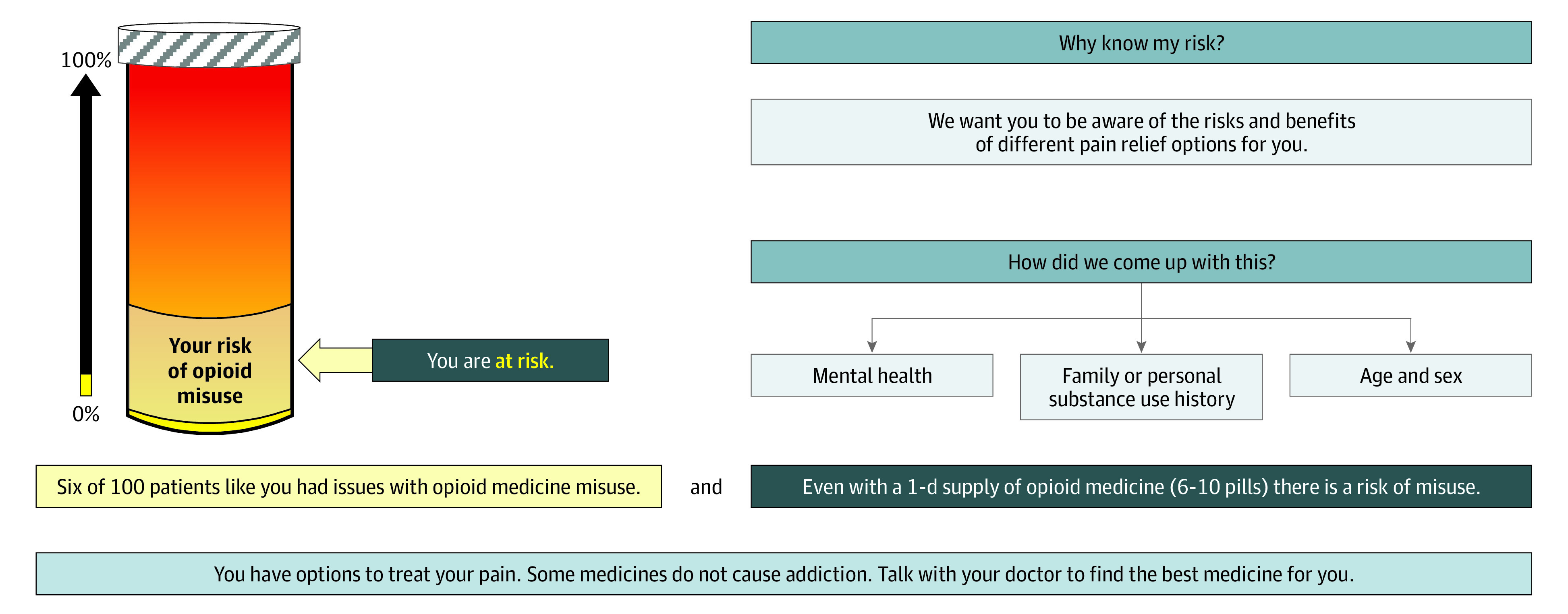
Opioid Risk Tool Indicating at Risk Adapted from a PowerPoint presentation in the supplement to Meisel et al.^16^

**Figure 2.**
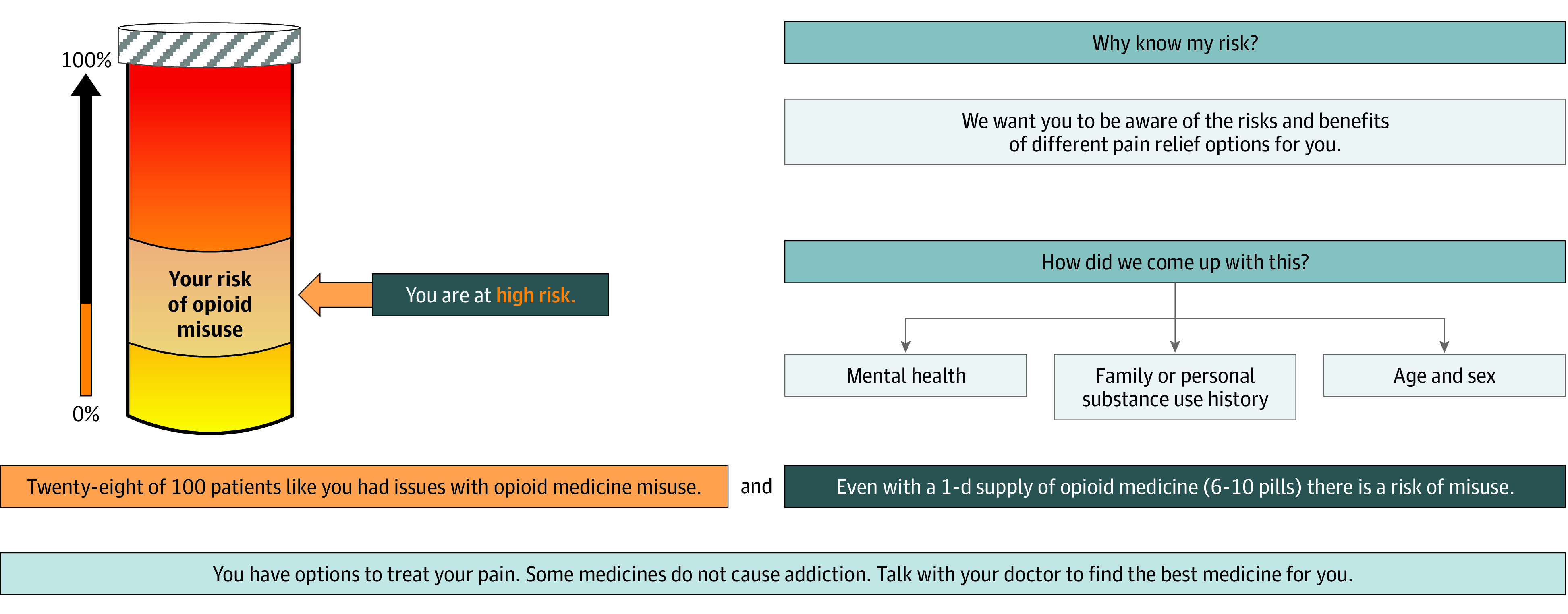
Opioid Risk Tool Indicating at High Risk Adapted from a PowerPoint presentation in the supplement to Meisel et al.^16^

**Figure 3.**
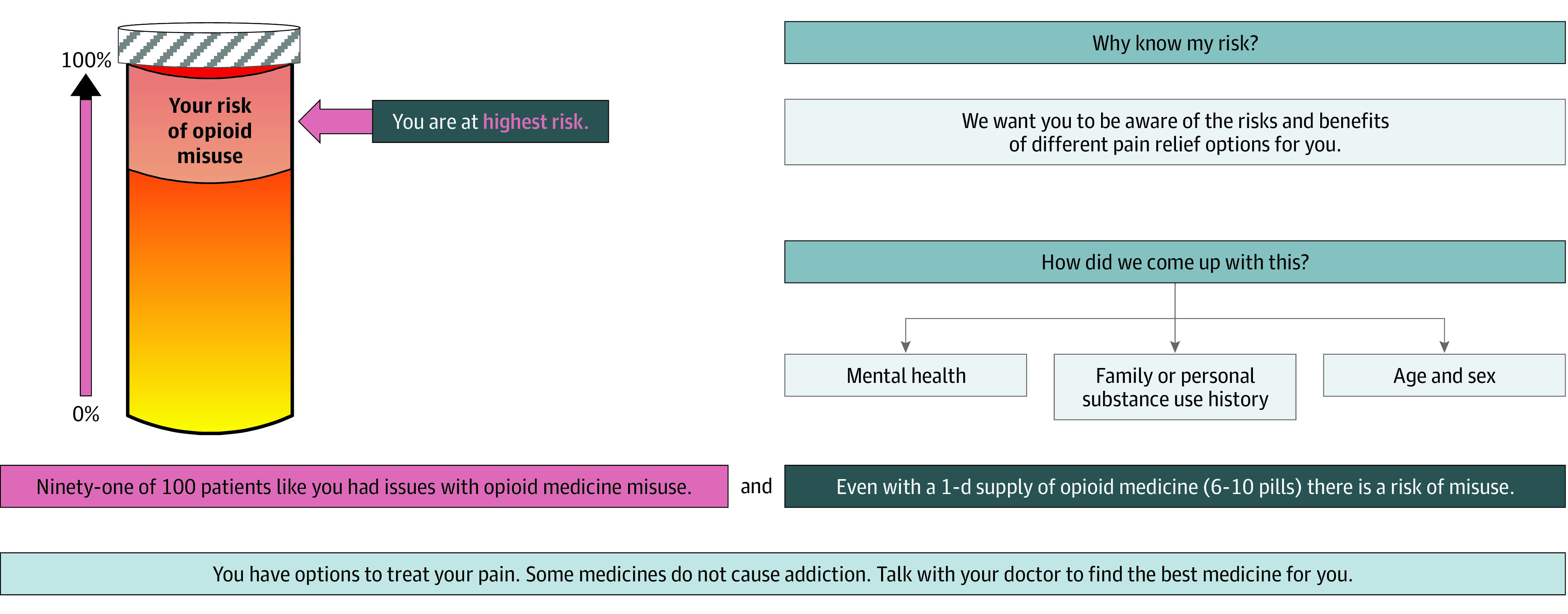
Opioid Risk Tool Indicating at Highest Risk Adapted from a PowerPoint presentation in the supplement to Meisel et al.^16^

In addition to the general risk information sheet, participants in the 2 intervention groups received a probabilistic risk tool that provided a visual representation of the participant’s risk of misusing opioids according to the Opioid Risk Tool (ORT).^15^ The ORT is a previously validated tool weighing a variety of clinical and experiential variables to determine risk of misusing opioids. Depending on the participants’ responses to the ORT, they could be categorized as at risk (Figure 1), at high risk (Figure 2), and at highest risk (Figure 3) for future opioid misuse.^16^

Participants in the third group received the narrative-enhanced probabilistic risk tool and were asked to watch narrative videos in addition to receiving the probabilistic risk tool. Participants viewed eight 1- to 3-minute videos of patients discussing their experiences with pain treatment and opioids (eAppendix and eTables 1 and 2 in the Supplement).

Hereafter, these 2 interventions will be referred to as the *risk interventions*. The full RCT protocol has been described elsewhere.^17^

### Qualitative Theoretic Framework

This qualitative study was informed by the Ottawa Decision Support Framework, which outlines the components, processes, and outcomes of a shared decision-making process.^18^ This framework includes a clear statement of decision options, presentation of evidence-based outcomes associated with alternative options, a value-clarification exercise, and coaching to encourage a shared decision-making conversation between patients and clinicians.^18^ Specifically, the interview guide and codebook were informed by the Ottawa Decision Support Framework. In addition, the Consolidated Criteria for Reporting Qualitative Research (COREQ) reporting guideline for qualitative research was used for study design and analysis of our data.^19^

### Sampling Strategy

Participants from the RCT were eligible to participate in the interviews if they received 1 of the 2 risk interventions; participants randomly assigned to receive usual care were not included because they did not receive one of the risk interventions and therefore could not reflect on them. The study team chose to interview participants who had already completed the RCT study events; in accordance with an institutional review board requirement, participants were eligible to participate if they had done so within the past year. Therefore, they completed the interviews between 3 months and 1 year after enrollment in the RCT.

Participants were recruited via telephone and email. Sampling was purposive to recruit trial participants with both back or neck pain and renal colic, across recruitment sites, at each ORT risk level, and from differing age and demographic groups. We anticipated that a sample of 30 to 40 participants would be needed to reach thematic saturation.^20^

### Development of the Interview Guide

An interview guide (eAppendix in the Supplement) was developed based on the Ottawa Decision Support Framework.^18^ The interview guide was developed by the research team, including 2 interviewers (A.R.D. and E.B.G.), the principal investigator, 2 coinvestigators, and the patient investigators who were community members serving on the research team. The guide was refined through an iterative process made to ensure it addressed the constructs in the Ottawa Decision Support Framework. The guide was piloted with initial interviewees (n = 3) and then refined.

### Conducting of Interviews

Interviews were conducted in English by A.R.D. and E.B.G., master’s-level researchers trained in qualitative research. At the interviews, A.R.D. was the research coordinator and E.B.G. was the project manager for the Life STORRIED trial. Interviews were conducted from June 4, 2019, to August 6, 2019, over the telephone and were audio recorded, transcribed, and edited to remove identifying information. An undergraduate research assistant listened to some interviews for training purposes. Participants were first given the opportunity to review the risk interventions that they received at RCT enrollment and were given a chance to react to them.

### Statistical Analysis

NVivo version 12.1 (QSR International) was used for analysis, which was conducted from August to December 2019. We conducted thematic analysis using a mixed inductive and deductive approach. The codebook was informed by the Ottawa Decision Support Framework and developed by the 2 interviewers along with the undergraduate research assistant. Each interviewer independently reviewed the first 5 transcripts, using open coding to create the first-round codes, and then both met to draft the first iteration of the study codebook. Definitions and criteria were determined for each code. The initial codebook was tested on 3 transcripts chosen at random, with 1 double coded by the interviewers, who then discussed how well the codes encapsulated the data. After additional refinement, the second iteration of the codebook was discussed among the team to ensure that the codes were consistent with the qualitative approach, the theoretic framework of the study, and the research question. There were 11 total nodes, with some having subnodes (eTable 1 in the Supplement).

The study team sent participants their transcribed interviews before coding to allow them to review and correct the content. Coding was conducted during a 3-month period after all interviews were complete. All coding was performed by the 2 interviewers, who had an intimate knowledge of the RCT objectives. To assess the level of coding agreement between the 2 coders, 20% of the transcripts were double coded, and a measure of agreement (the Cohen κ coefficient) was calculated. For any code with κ < 0.90, the researchers reviewed their codes, discussed discrepancies, and agreed on subsequent coding. Thematic analysis was conducted by the full research team, who met biweekly to develop code summaries and discuss emerging themes.

## Results

Two hundred sixty participants from the Life STORRIED trial met criteria for participation in this qualitative study. Of the 181 participants from the RCT who were contacted to participate in an interview, 36 agreed. The median age was 38 years (range, 21-67 years), 22 individuals were female (61%), 14 were male (39%), 2 were Asian (6%), 14 were Black or African American (39%), 5 were Hispanic or Latino/Latina/Latinx (14%), 14 were White (39%), and 6 were other races (17%) (includes American Indian or Alaska Native and Native Hawaiian or Other Pacific Islander; “other” was listed as an option that participants could choose). According to the ORT, 27 participants (75%) were at risk, 7 (19%) were at high risk, and 2 (6%) were at highest risk. Participants’ baseline characteristics are presented in Table 1. The interviews lasted on average 20 to 30 minutes.

**Table 1.** Characteristics of Participants

Characteristic	Participants, No (%) (N = 36)
Age, y	
Median (range)	38 (21-67)
20-29	6 (17)
30-39	12 (33)
40-49	8 (22)
50-59	6 (17)
60-69	4 (11)
Sex	
Female	22 (61)
Male	14 (39)
Race	
Asian	2 (6)
Black or African American	14 (39)
White	14 (39)
Other or multiracial^a^	6 (17)
Ethnicity	
Hispanic or Latino/Latina/Latinx	5 (14)
Non-Hispanic	31 (86)
Condition	
Back or neck pain	25 (69)
Kidney stone	11 (31)
Arm	
PRT	18 (50)
NE-PRT	18 (50)
ORT score	
At risk	27 (75)
At high risk	7 (19)
At highest risk	2 (6)
Opioid prescription at discharge	
Yes	12 (33)
No	24 (67)

Abbreviations: NE-PRT, narrative-enhanced PRT; ORT, Opioid Risk Tool; PRT, probabilistic risk tool.

^a^ Includes American Indian or Alaska Native and Native Hawaiian or Other Pacific Islander. “Other” was also listed as an option that participants could choose.

Five major themes emerged, reflecting the experiences and perceptions of participants as they discussed analgesic-related preferences and decisions made during their visit. The focus of these 5 themes was the factors associated with the risk interventions; clinician paternalism; analgesia attributes and previous experiences; individual self-identity, attitudes, and values; and perceptions of clinician bias. There were no thematic differences found between the 2 groups.

### Theme 1: Factors Associated With Risk Interventions

The perceived influence of the interventions on participants’ stated risk perceptions and decisions and preferences varied. Overall, participants found the interventions to be poignant, and many participants accurately recalled themes from both the probabilistic risk tool and the narrative videos. One participant was “alarmed” by her risk score because she learned she was at high risk (Table 2, quote 1).

**Table 2.** Key Representative Quotes for Each Theme

Theme	Quote
Theme 1: factors associated with risk interventions (they conveyed risk but did not always change individual risk perceptions)	Quote 1: “I think she [the research assistant] … asked me questions and then she gave me a percentage as to how I would be as an opioid user if I were to use it … . That kind of alarmed me, too, because I was like, wow. I’m at a high risk for opioids?” (probabilistic risk intervention, at high risk) Quote 2: “I had no idea that opioids could be so addictive. That actually … compounded my, oh, I don’t want to do the pills thing because they may not be the most natural way to go about it … I had no idea that they were so addictive, so actually after watching those videos it scared me away from them a lot more than I was before. Before I saw it as like the last resort and now I kind of still see it as the last resort but kind of like I’ve really got to be desperate before I go there.” (probabilistic risk intervention, at risk) Quote 3: “I know she [the research assistant conducting enrollment] was talking to me about people can get pretty much addicted to the pain medication that they give you. But, like I say, I don’t even take medication, so I can’t get addicted to something that I don’t like taking.” (narrative intervention, at highest risk)
Theme 2: clinician paternalism (clinician-led decision-making was often described)	Quote 4: “He didn’t really talk to me about options, nothing regarding any opioids, nothing like that. We both felt like the pain wasn’t so bad where I would need to go to that extent. I think which is why we decided on just sticking to over-the-counter medication.” (narrative intervention)
Theme 3: analgesia attributes and previous experiences (how they drove treatment preferences)	Quote 5: “It was just that it [opioid medication] makes me so sick … because of that I just automatically disregard the choice of using it.” (narrative intervention) Quote 6: “I don’t do drugs or anything like that. And I don’t wanna get dependent on anything. So that’s why I did not want that [prescribed opioid medication] long term and I was happy about the pain subsiding.” (probabilistic risk intervention, at risk)
Theme 4: individual self-identity, attitudes, and values (how they guide pain management)	Quote 7: “We’re more holistic in the family and my wife has turned me that way. We’re a little more—we really try to avoid medicines unless it’s absolutely necessary, antibiotics and things like that … . Well I think that naturally there’s a lot of things you can do to benefit yourself, mentally and physically. And I don’t want to just jump to medicine as the first solution, I don’t think it should ever be the first solution. For instance, a coworker has high cholesterol, so they put him on medication. Maybe his first thought should have been changing diet or exercise, or a combination of both. And I find that a combination of diet and exercise will probably reduce and relieve a lot of symptoms that people are feeling.” (probabilistic risk intervention, at risk)
Theme 5: perceptions of clinician bias	Quote 8: “I felt that the doctor did not listen to me, and I guess I felt that the doctor didn’t feel that the pain was as bad as it was, and even though I told her that my surgeon had told me in the past that if I ever had this again, that it would probably need surgery, but again she didn't listen to me.” (narrative intervention, at risk) Quote 9: “They didn’t believe that I was unable to walk or like maybe they thought that I could walk and I didn’t want to walk, but I really couldn’t walk.” (probabilistic risk intervention, at risk) Quote 10: “I don’t know. I was just so, so disappointed by the care that I received. And the person who treated me made me feel like I was drug seeking or something … I felt like he just didn’t take my pain seriously, I guess.” (narrative intervention, at high risk) Quote 11: “And of course—and I don’t want to sound racist, but I thought—they say, oh maybe it’s some kind of venereal disease. And I say, no. I thought it was a urinary tract infection … . And I kept telling them, I’m in so much pain. It’s just like, you’re making too much noise. You’re yelling. You need to stop making all of that noise. She was very rude … I think that the lady was talking—the nurse was talking down to me … I think that—there’s a lot of documentation showing that White females are given more—are taken serious [*sic*] when you’re in pain over Black females.” (narrative intervention, at risk)

Some participants definitively stated the narrative videos influenced their preference not to take opioid medications: “For me, after watching those videos, I was like well, if I don’t think I need it, I don’t really wanna run the risk of maybe having to—[not know] … whether or not I would be addicted.” One participant discussed how the narrative videos informed her views on pain treatment and the addiction potential of opioids (Table 2, quote 2).

Other participants did not always integrate the messages of the risk interventions into their understanding of their own personal risk. One participant found the narrative videos powerful but not personally relevant: “I felt like, while it was also really sad and emotional, I felt like it, I guess it didn’t apply to me, personally.” Similarly, 1 participant, despite receiving an ORT score of highest risk, disregarded the probabilistic risk tool intervention because she held preconceived ideas about pain medication and opioids (Table 2, quote 3).

Nearly all participants indicated engagement with the risk interventions, recalling important themes from them. Despite this engagement, these themes were not always explicitly incorporated into the pain treatment decisions as described by participants.

### Theme 2: Clinician Paternalism

Some participants indicated that their treatment decisions were based solely on what their clinicians decided. A few participants referred to conversations with their clinicians that led to a shared decision, specifically using “we” language when discussing the decision (Table 2, quote 4). The participants most often indicated that they followed what the clinicians instructed.

Some participants indicated that their clinicians’ prescribing decisions did not align with their preferences. For example, when asked whether pain treatment options were discussed with their clinician, 1 participant replied that they were not and then went on to explain they never took the medication because “I’ve been on them … it made me druggy drowsy and I can’t function.”

### Theme 3: Analgesia Attributes and Previous Experiences

In many cases, previous experiences with medication appeared to outweigh the risk information received from the risk interventions. Some patients described negative experiences, such as nausea, with certain medications that heavily influenced their medication decisions (Table 2, quote 5).

Specific attributes of pain medication options were also discussed. A medication having few adverse effects was cited as a reason for participants’ choosing to take it: “… it didn’t have that much side effects [*sic*] from some of the other pain killer, I decided to go with the ibuprofen.” Risk of addiction was a reason participants chose not to take opioids in particular (Table 2, quote 6). These participant responses demonstrate that patients do value risk information when considering pain treatment options.

### Theme 4: Individual Self-identity, Attitudes, and Values

Many individuals indicated that their self-identity influenced how they treated their pain. This self-identity influenced their treatment decisions as well as how they understood the interventions. For example, one participant said, “I’m not a pill popper.” When reflecting on the ORT, another participant said plainly, “I don’t do pills and needles.”

Some participants described their personal values regarding pain treatment and conveyed that they generally avoid taking medication: “I mean … I never did take over-the-counter medicine or prescriptions that was [*sic*] given to me … I really don’t even like taking medicine.” Participants mentioned their desire to seek alternative pain treatment, including holistic medicine and marijuana, and highlighted the importance of lifestyle changes, including diet and exercise (Table 2, quote 7). Personal values were predetermined and often strong factors associated with the development of preferences for pain management.

### Theme 5: Perceptions of Clinician Bias

Perceptions of clinician bias and discrimination were associated with the care and treatment received by some of the participants. All participants who described an experience of discrimination identified themselves as women. A striking theme reflected occurrences in which the pain that the participants were experiencing was not being taken seriously by their clinicians. This attitude on the part of the clinicians was noted during attempts to communicate health history (Table 2, quote 8), with 1 participant being asked to prove her inability to walk (Table 2, quote 9). Two participants believed that they were being treated as if they were seeking drugs (Table 2, quote 10).

One participant, in particular, described the racial discrimination experienced when pursuing treatment for symptoms (Table 2, quote 11). This participant visited several EDs before finally receiving adequate treatment at the enrollment site of the RCT.

These participants described how the discrimination resulted in poor communication with their care team. This lack of communication played a part in a decision-making process that was ineffective and resulted in inadequate care.

## Discussion

An ED visit for an acutely painful condition can be a disorienting experience for a patient. Providing accurate and engaging information in this environment can be challenging. Our qualitative study allowed for participants receiving 2 opioid risk interventions to reflect on their experiences and provide insight into their decision-making processes regarding their pain treatment. Most participants described their experiences with the interventions as engaging, often describing the ways that they were surprised by the content presented. Some participants explicitly stated that the interventions changed their opinion on opioid medications or influenced their decisions. However, others described alternate factors that seemed to inform the decision-making process, including deference to their clinician, previous experiences with pain medication and attributes of those medications, personal values and self-identity, and experiences of bias and discrimination.

Although the risk communication interventions were engaging for many patients, they were not always cited by participants as an integral part of the decision-making process for pain treatment. Furthermore, some participants struggled to understand how the interventions were personally relevant because they believed their prior experiences rendered the additional risk information less important. Availability bias (believing that recent personal experiences outweigh current information) or optimism bias (believing oneself to be less susceptible to harms than the general population) may have contributed to these considerations.^21,22^

Altogether, these results provide insight into the quantitative results from the trial published previously.^12^ One of the primary outcomes of the RCT was ability to accurately recall one’s risk score after receiving the risk tool; overall, rates of accurate recall for both risk intervention groups were low (38.8% for those who received the probabilistic risk tool alone and 43.7% for those who received the narrative-enhanced probabilistic risk tool).^12^ A possible explanation for these low rates of recall could be that self-identity—for example, identifying as someone who does not “do pills”—renders risk information less relevant. Another outcome from the RCT was that the risk interventions led to high levels of satisfaction and perceived shared decision-making; these quantitative results align with our qualitative findings of participant engagement with the risk communication interventions.

### Limitations

There are limitations to this study. First, because this was a qualitative study, the generalizability of our results is limited. However, the study was conducted within the context of an RCT. All participants therefore met similar eligibility criteria and had a consistent experience when exposed to the interventions, strengthening the internal validity of our findings. Furthermore, purposive sampling provided diversity in age, sex, race, and ethnicity. Second, because some interviews were conducted several months after participant enrollment in the RCT, they could be subject to recall bias. It is also possible that self-selection played a role because participants who experienced discrimination may have been more likely to participate. Third, bias could have been introduced by the interviewers because they were familiar with the RCT objectives and had themselves conducted the enrollment. The interviewers attempted to mitigate this by closely following the interview guide and asking open-ended questions that did not lead participants to provide certain responses.

## Conclusions

In this qualitative study, patient decision-making for pain treatment in the ED was associated with clinician opinion, patients’ previous experiences with pain treatment, their values and perceptions of self, and their experiences of bias. Most participants commented on the powerful lessons that they learned from the risk interventions. Although some participants explicitly incorporated information into their decision-making, others did not state that the interventions influenced their medication preferences. Some patients may have experienced availability bias or optimism bias, and future research should investigate how to overcome these biases when patients are told about risk. In addition, the discrimination experienced by some of the female participants further underscores the need for future research into decision aids and communication tools that center the patient’s experience and decrease opportunities for potential clinician bias to interfere with the therapeutic relationship.

## Notes

Supplement. **eAppendix.** Life STORRIED Qualitative Aim Interview Guide **eTable 1.** Life STORRIED Codebook **eTable 2.** Narrative Videos Featured in Narrative-Enhanced Probabilistic Risk Tool
