# Supplementary material for: Patient Perceptions About Opioid Risk Communications Within the Context of a Randomized Clinical Trial
Source: JAMA Netw Open. 2022 Aug 18;5(8):e2227650. doi: 10.1001/jamanetworkopen.2022.27650 (PMC9389346; doi:10.1001/jamanetworkopen.2022.27650)
Supplement: Supplement. — eAppendix. Life STORRIED Qualitative Aim Interview Guide eTable 1. Life STORRIED Codebook eTable 2. Narrative Videos Featured in Narrative-Enhanced Probabilistic Risk Tool [file jamanetwopen-e2227650-s001.pdf]

Supplementary Online Content

Dolan AR, Goldberg EB, Cannuscio CC, et al. Patient perceptions about opioid risk communications within the context of a randomized clinical trial. *JAMA Netw Open*. 2022;5(8):e2227650.
doi:10.1001/jamanetworkopen.2022.27650

eAppendix. Life STORRIED Qualitative Aim Interview Guide
eTable 1. Life STORRIED Codebook
eTable 2. Narrative Videos Featured in Narrative-Enhanced Probabilistic Risk Tool

This supplemental material has been provided by the authors to give readers additional information about their work.

© 2022 Dolan AR et al. *JAMA Network Open.*

eAppendix. Life STORRIED Qualitative Aim Interview Guide
We will assess how the Life STORRIED subjects --randomized to the narrative and probabilistic only intervention arms -- perceive, share, use, and recall the communication tool and incorporate them into preferences and understanding about pain treatment.
Two of the goals of the narrative and probabilistic interventions are to 1) provide evidence-based risk information in a format that minimizes the cognitive burden of comparing and weighing attributes between treatments, and 2) encourage a conversation and shared decision making process between the patient and provider.
Goal 1: Understand how subjects perceived, contextualized (through their own experiences), and used (in their own words) the narratives and probabilistic risk tool towards these goals.
Goal 2: Describe the range of potential explanations that underpin the ultimate findings of the comparative effectiveness trial across the primary and secondary outcomes.
Script for first call:

Hi my name is _____, I am calling from* (site) *with the Life STORRIED study*[1]*, may I please speak with* (participant name)? *I am calling about the study you enrolled in a few months ago when you came to the ER for* (back pain/kidney stone pain). *You enrolled in the Life STORRIED study and agreed to be contacted after the study was complete. I was wondering if I could ask you a few follow-up questions about your experiences with your pain and pain treatment. I would like to conduct a 20-30 min interview with you about the time you went to the emergency room to treat your pain and you enrolled in this study. The purpose of these interviews is to help us understand how to best improve patient and doctor communication. If you are interested, we could set up a time for me to call you. The interview will be recorded for quality assurance purposes and you will receive 20 dollars for your participation. Everything you say will be confidential and your anonymity will be protected.

***Do you still have the ClinCard that was given to you when you enrolled in the study? If not, we can send you another card* (to be figured out at the end of the call after the interview). (Schedule the interview)**

Additionally, we will be sending you some of the information we shared with you the day before your interview (risk tool image for arm 2 and risk tool image plus videos for arm 3). Please be on the lookout for that and we will talk to you soon. Thank you.

Script for calling during scheduled interview:

Hi this is ______, calling from the Life STORRIED study. May I please speak with* (participant name)? *I am calling for our scheduled interview. Does this time still work for you?* (If yes) *Great, thank you so much for your participation. As a reminder, the purpose of these interviews is to help us understand how to best improve patient and doctor communication. The interview will be recorded for quality assurance purposes and you will receive 20 dollars for your participation. Everything you say will be confidential and your anonymity will be protected. Do you agree to participate?

(If yes) *Great, I am going to turn on the recorder and we can begin.*

General Questions

Thinking about the time before we met you in the ER:

1. What brought you to the ER on the day you were enrolled in the study?

2. Tell me about your experience with low back pain (or) kidney stones [depending on the patient] prior to the visit when we met you in the ER.

3. Before you came for care, how did you hope your pain would be managed?
    i. Prompt: Did you know, ahead of time, that you would want pain medication?

Okay, now we're going to transition to talk about the day that you were enrolled in the study:

© 2022 Dolan AR et al. *JAMA Network Open.*

4. Tell me about the treatment and/or care you received at the hospital.
    a. Probe if applicable- Why do you think you didn't get the attention you deserved?

5. Describe how your doctor talked to you about your options for managing your pain.
        i. How did you feel about that interaction? Was there anything else you hoped the doctor would do [or ask]?

6. Overall, was there anything about your pain treatment you wish you would have talked to your doctor about that you didn't? If so, why didn't you have that conversation?

7. What medications were you prescribed before leaving? Any medications specifically for managing pain?
    a. If yes:
        i. What medication were you prescribed?

        ii. Who decided what medication you would take? Did you discuss these options with the treating physician?

        iii. Tell me about your experience with this medication. Are you still taking it? How do you feel about the medication?

    b. If no:
        i. Tell me more about that. Did you choose not to take prescription pain medication for this pain? If so, why did you make this decision?

8. What has your experience with pain been like since the ER visit?

9. If you had known then what you know now, what would you have done differently in the management of your pain? Would you have chosen the same or a different way to treat your pain?

10. How have your thoughts changed about how to treat your pain?

11. What have you learned about pain treatment specifically from this study experience that you didn't know before?

12. What do you remember about the sheet that told you your risk of having an issue with opioid medication misuse?

13. What was your experience like having someone use this sheet to explain your risk of having an issue with opioid medication misuse?
        i. What would you change about the way this sheet was discussed with you while you were in the Emergency Department?

14. [If they are Arm 3, wait to ask this question until the end] Tell us about the discussion you had with your doctor(s) after we left you in the room?
        i. What was your discussion around pain treatment options?
        ii. What was discussed in regards to the tools we shared with you? (risk images)

Subjects in the Narrative Arm

1. Which videos did you watch in the Emergency Department? What do you remember about them?

2. Did you watch any videos after you left the Emergency Department? Which ones?

3. What about the stories of the people in the videos were believable or relatable?

4. What was your experience like learning about the stories of these individuals while you were in the Emergency Department?

5. Did you talk to anyone else about the videos that you watched? Did you talk to your provider about them? If so, what was that conversation like?

6. What would you change about the way the videos were shown to you while you were in the Emergency Department?

7. Tell us about the discussion you had with your doctor(s) after we left you in the room?
    i. What was your discussion around pain treatment options?
    ii. What was discussed in regards to the tools we shared with you? (narratives and risk images)

© 2022 Dolan AR et al. *JAMA Network Open.*

eTable 1. Life STORRIED Codebook

	Node	*Definition*	*When NOT to use*	*Examples to be included later*	*Sub-nodes*
**1**	Reason for visit	Use this node when participants discuss *specific* pain that brought them into the ED		R: I had excruciating pain in my left side, my back, my chest. And it just came up on me and I didn't know where it come from.	Back Pain / Kidney Stone Pain / Other
**2**	Previous Experiences with Condition	Use this node when participants discuss previous instances of back or kidney stone pain		R: This last stone that I had prior to that, which was probably 20 years before I first had the first diagnosis	Back Pain / Kidney Stone Pain / Other
**3**	Expectations of Visit	Use this node when participants discuss expectations about treatment, pain management, etc.		R: Oh, I didn't care. I just knew I was in the hospital and they was gonna make it right<br><br>R: I didn't have much of an expectation, just because I think a lot of the pain was just kind of – made me think of pain	Pain Management/Non-Pain Management

© 2022 Dolan AR et al. *JAMA Network Open.*

				more than anything else.	
**4**	Medical Visit Experience	Use this node when participants talk about how they felt the visit went and what happened<br><br>Also<br><br>**Use this node when participants discuss any conversations around treatment plan, explanation of diagnosis, pain treatment discussion**		R: But once I got to the back, they gave me that medicine. It took a while for it – it seemed like a while for it to work. And eventually I – after they admitted me on paper, then I passed the kidney stone<br><br>R: And of course – and I don't want to sound racist, but I thought – they say, oh maybe it's some kind of venereal disease	Patient Satisfaction/ Patient Dissatisfaction/ Concordance with Treatment Plan/Communication with care team
**5**	Decision Making	Use this node when any the patient makes reference to medication decisions or treatment decisions.		R: They did explain about different, different pain killer. They explained to me the side effects and other stuff. That's how I decided to go with the ibuprofen and the patch.	

© 2022 Dolan AR et al. *JAMA Network Open.*

**6**	Attitudes toward Pain Management	Use this node when participants talk about reflections about pain management		R: Yeah, I probably would've done the same thing, just because – yeah I would've probably done the same thing back then as – what I know now	Specifically not going to sub node- may break down into themes for the paper- individual vs. societal
**7**	Risk Tool Experience	Use this node when participants discuss risk tool assessment, having someone explain the sheet, etc.		R: I think that the person who came in did a really good job of explaining the information that was on the form	Positive Attitude toward Risk-Assessment Tool / Negative Attitude toward Risk-Assessment Tool/Risk Tool Recall/
**8**	Video Experience	Use this node when participants discuss their experience viewing videos		R: I think one that I saw that made a message that this could honestly happen to anybody, you know. That was really what I took from that.	Positive Attitude toward Videos / Negative Attitude toward Videos/Video Recall
**9**	Post-Study Tool Conversation with Provider	Use this node when participants discuss speaking with their provider after study experience		R: I don't think so. I don't think we discussed anything as far as pain meds	Post-Intervention Conversation with Provider /Treatment Plan
**10**	Post-Visit	Use this node when participants discuss any changes in decisions made about pain management		**I: And why did you decide to stop taking the medicine?**	Experience with Pain and Pain Management/Condition Management/Reflections on their care

© 2022 Dolan AR et al. *JAMA Network Open.*

		after study experience, and by whom (such as the rationale for taking a certain medication, etc.) Also can discuss Pain Experience since ED visit		R: I think just the pain was just – it was more manageable. I was able to do my every day functions without being in too much discomfort<br><br>R: I just kind of beared through it just because doing – right after that, I was back to work and stuff like that	
**11**	Study Experience	Use this node when participants discuss experiences about the study			Positive Study Experience/Negative Study Experience/Lessons Learned

© 2022 Dolan AR et al. *JAMA Network Open.*

eTable 2. Narrative Videos Featured in Narrative-Enhanced Probabilistic Risk Tool

Name	Description
Paul	Paul misused opioids after being prescribed Oxycontin for a painful condition.
Linda	Linda, a nurse, misused opioids after surgery for cancer and migraine headaches.
Elise	Elise lost her young daughter to a heroin overdose after being introduced to opioids by friends.
Mike	Mike prefers to avoid opioids due to his family history of addiction.
Jeff	Jeff, a nurse, misused opioids after being prescribed them for a broken arm.
Dena	Dena's doctor, knowing her history of addiction, helped her to avoid misusing opioids after a painful cancer surgery.
Sharon	Sharon experiences chronic pain and takes opioids daily to cope with the pain.
Rachel	Rachel lost her brother to an opioid overdose after he was introduced to them by a friend.

© 2022 Dolan AR et al. *JAMA Network Open.*
